# Effect of Commercial Yeast Starter Cultures on Cabernet Sauvignon Wine Aroma Compounds and Microbiota

**DOI:** 10.3390/foods11121725

**Published:** 2022-06-13

**Authors:** Meiqi Wang, Jiarong Wang, Jiawei Chen, Christian Philipp, Xiaoning Zhao, Jie Wang, Yaqiong Liu, Ran Suo

**Affiliations:** 1 College of Food Science and Technology, Hebei Agricultural University, Baoding 071001, China; wmq25802022@163.com (M.W.); jrwang1031@163.com (J.W.); wangjie@hebau.edu.cn (J.W.); ransuo@hebau.edu.cn (R.S.); 2 China Great Wall Wine Co., Ltd., Zhangjiakou 075400, China; jiajia6873@163.com (J.C.); zhaoxiaoning2013@sina.com (X.Z.); 3 Höhere Bundeslehranstalt und Bundesamt für Wein- und Obstbau, Wienerstraße 74, 3400 Klosterneuburg, Austria; christian.philipp@weinobst.at

**Keywords:** Cabernet Sauvignon, wine yeast, volatile composition, microbial community

## Abstract

Commercial Saccharomyces cerevisiae plays an important role in the traditional winemaking industry. In this study, the correlation of microbial community and aroma compound in the process of alcohol fermentation of Cabernet Sauvignon by four different commercial starters was investigated. The results showed that there was no significant difference in the fermentation parameters of the four starters, but there were differences in microbial diversity among the different starters. The wine samples fermented by CEC01 had higher microbial abundance. GC-MS detected a total of 58 aromatic compounds from the fermentation process by the experimental yeasts. There were 25 compounds in the F6d variant, which was higher than in other samples. The PCA score plot showed that 796 and F15 yeast-fermented wines had similar aromatic characteristic compositions. According to partial least squares (PLS, VIP > 1.0) analysis and Spearman’s correlation analysis, 11, 8, 8 and 10 microbial genera were identified as core microorganisms in the fermentation of 796, CEC01, CECA and F15 starter, respectively. Among them, *Leuconostoc*, *Lactobacillus*, *Sphingomonas* and *Pseudomonas* played an important role in the formation of aroma compounds such as Ethyl caprylate, Ethyl caprate and Ethyl-9-decenoate. These results can help us to have a better understanding of the effects of microorganisms on wine aroma and provide a theoretical basis for improving the flavor quality of Cabernet Sauvignon wine.

## 1. Introduction

Commercial yeast starter cultures are widely used in large-scale winemaking and provide far more reliability for vinification [1]. However, different strains of Saccharomyces cerevisiae can produce significantly different flavor profiles when fermenting the same must [2]. This is a consequence of both the variable ability of Saccharomyces wine yeasts to release varietal aroma compounds from grape aroma precursors and the differential capacity to synthesize yeast-derived volatile compounds de novo [3,4]. Large-scale wine fermentations are never sterile, which leads to the microbial diversity in grape must, in which many species of yeasts, bacteria, and fungi come from grape skin and the vineyard environment. Microbial species, relative abundance and interaction play important roles in fermentation, determining the final quality and sensory characteristics of wine products [3,4,5]. In previous studies, investigation of the microbial communities existing in vineyards, the winery environment, grape berry surface and spontaneous fermentation process by molecular means was carried out [6,7]. However, more information is required to help establish the role of commercial yeasts and their effect on wine production volatiles. Exploring the microbial succession and the determination of core microorganisms in the process of commercial starter inoculation fermentation will help us to better understand the quality control of wine.

Aroma is an important index to evaluate wine products. A successfully made wine should have pleasant and distinctive flavor characteristics, which are caused by the combined action of various aroma compounds [8]. At present, more than 1300 volatile compounds, including esters, alcohols, acids, carbonyl and sulfur compounds [9], have been detected in wine. About 400 volatile compounds come from wine fermentation, which is performed by microorganisms. The volatile aroma of wine comes from the following sources: the grape variety itself; specific production process steps, such as cold maceration; metabolites from yeast and bacteria during fermentation; and chemical reactions from the storage process [8]. The combination of variety aroma and fermented aroma is an important factor to distinguish specific winemaking areas [9]. Volatile compounds in wine influence the sensory experience of consumers, and at the same time help them to understand the style characteristics of wine in specific producing areas.

Cabernet Sauvignon, as one of the most widely planted red wine grape varieties in the world, is also widely planted and used by winemakers in China. It can produce red wine with deep color, rich flavor and heavy tannin, owing to the characteristics of high pigment, high acid, high tannin and rich aroma [10]. Shacheng, located in Zhangjiakou City, Hebei Province, is a major wine-producing area in China. This area has long sunshine time, a large temperature difference between day and night, and little rainfall, which is very beneficial to the growth of grapes, and its unique sandy soil is also conducive to grape nutrient storage. Advantaged geographical conditions produce high-quality Cabernet Sauvignon. However, there are few studies on the aroma composition and effect of fermentation microorganisms in the alcohol fermentation process of Cabernet Sauvignon in the area.

In this study, we explored the effects of four starters on the volatile aroma and microbial succession of Cabernet Sauvignon wine. The next generation high-throughput sequencing technology was used to monitor the dynamic changes in the microbial community structure of wine samples inoculated with different starters. Identification of core microorganisms in different starters was established using the PLS-DA model. Aroma compounds were analyzed by headspace-solid-phase microextraction (HS-SPME) combined with gas chromatography-mass spectrometry (GC-MS). Based on this, we evaluated the effects of the core microorganisms on the sensory quality of wine and discussed the potential correlation between microorganisms and aroma compounds combined with the Spearman coefficient.

## 2. Materials and Methods

### 2.1. Industrial Fermentation

The grapes (Cabernet Sauvignon) were harvested at optimal technological maturity (total sugars: 221–222 g/L glucose; total acidity: 6–7 g/L tartaric acid) on 11 October 2020 in a vineyard of the Shacheng region, NE China, and were used as material for fermentation. Vinification was also carried out on an industrial scale at China Great Wall Wine Co., Ltd. Pectinase (0.04 g/kg, Lafase He Grand Cru) and potassium metabisulfite (80 mg/L) were added into the must after crushing, and the loading volume was approximately 75–80% of the 90 m^3^ tank capacity. Four kinds of commercial active dry yeasts (Saccharomyces cerevisiae, 0.25 g/L, Zymaflore F15 from LAFFORT Co., Bordeaux, France; 796 from Maurivin Yeast Co., North Ryde, NSW, Australia; CECA and CEC01 from Angel Yeast Co., Yichang, China) were rehydrated with water (1:10) and transferred to must for alcohol fermentation. Grape musts were fermented at a controlled temperature (≤26 °C) with pumping over three times per day. Alcohol fermentation was considered finished when the residual sugar was below 2 g/L. All fermentations were carried out twice. Samples (500 mL) were collected from grape juice (day 0), early stage (day 2, residual sugar remaining 70%), middle stage (day 4, residual sugar remaining 30%) and late stage (day 6, residual sugar less than 10%) of the industrial must fermentation used for microbial determination. Each sample was centrifuged (8000 r/min, 10 min) at 4 °C, and the precipitates were subjected to high-throughput sequencing (HTS). All samples were stored at −80 °C until analysis. Each sample was analyzed in triplicate. All the acronyms used for the samples and their descriptions are in Table 1.

### 2.2. Physicochemical Analysis

During fermentation, changes in several oenological parameters, including pH and ethanol, total sugar and total acid contents, were monitored by Fourier Transform Infrared Spectrophotometer (Wine Scan FT120, FOSS A/S, Hilleroed, Denmark) through a 32 μm path length cuvette from 926 to 5012 cm^−1^ at 4 cm^−1^ intervals. Ten interferograms were averaged to produce each infrared spectrum. The Wine Scan was configured to acquire duplicate infrared spectra for each sample. Wave number (cm^−1^) = 3.858 × pin number. The number of scans generated by each sample, selection of wavenumber, processing of the spectrum, and statistical data analysis settings were determined by the manufacturer and could not be changed by the user.

### 2.3. Microbiological Analysis

A 40 mL sample of fermentation juice was taken, centrifuged at 8000 RPM for 10 min, then the Fast DNA SPIN Kit (MP Biomedicals, Irvine, CA, USA) was applied to extract the total DNA genome. DNA integrity was detected by gel electrophoresis using 1% agarose gel at a voltage of 5 V/cm for 20 min. High-quality DNA was selected for PCR amplification and HTS. The PCR procedure was as listed: Bacterial 16S r RNA gene sequences were amplified using the universal primer pair 338F (5′-ACTCCTACGGGAGGCAGCAG-3′) and 806R (5′-GGACTACHVGGGTWTCTAAT-3′), and the PCR primers targeting ITS region were ITS1F (5′-CTTGGTCATTTAGAGGAAGTAA-3′) and ITS2R (5′-GCTGCGTTCTTCATCGATGC-3′). Reaction conditions were 95 °C pre-degeneration for 3 min; 95 °C degeneration for 30 s; 55 °C annealing for 30 s; 72 °C extension for 45 s, repeat 35 (fungal) and 27 cycles (bacterial); 72 °C complementary extension for a final 10 mins; reaction system was compelling of 20 μL, including 4 μL of 5 × Fast Pfu buffer, 2 μL of dNTP (2.5 mM), 0.8 μL of forward primer (5 μM), 0.8 μL of reverse primer (5 μM), 0.4 μL of Fast Pfu polymerase, 0.2 μL of BSA, 10 ng of template DNA, and double-distilled water. The PCR products were analyzed by electrophoresis on 2% agarose gels. The PCR products were purified with an Axy Prep DNA Gel Extraction Kit (AXYGEN Biosciences, San Francisco, CA, USA).

Sequencing libraries were sequenced on an Illumina Miseq platform at Majorbio Bio-Pharm Technology Co., Ltd., Shanghai, China. The optimized sequence was clustered into operational taxonomic units (OTUs; defined by 97% similarity) using USEARCH software. The valid sequences in the samples were compared with the Silva database.

### 2.4. Volatile Compound Analysis

The aroma compounds in the samples were extracted by headspace solid-phase micro-extraction (HS-SPME) and detected through GC-MS (7890B–5977 A, Agilent, Palo Alto, CA, USA). Wine samples (7.5 mL) were pipetted into 15 mL SPME glass vials containing 1 g of sodium chloride (Sinopharm Chemical Reagent Co., Ltd., Shanghai, China), and then 15 μL of an internal standard solution (3-octanol, 300 mg/mL, Sigma–Aldrich, St. Louis, MO, USA) was added. The vials were incubated in a water bath at 40 °C for 15 min before exposing the DVB/CAR/PDMS fiber (50/30 μm, Supelco, Inc., Bellefonte, PA, USA) for 40 min at 40 °C. Desorption of volatiles was effected at 230 °C for in the injection port of GC-MS system (7890 B-5977 A, Agilent, Palo Alto, CA, USA). Compounds were separated on a HP-5MS column (60 m × 0.25 mm i.d., 0.25 μm df; J&W Science, Agilent, USA) using helium as the carrier gas at a constant flow rate of 1 mL/min. During analysis, the oven was kept at 50 °C for 2 min, then the temperature was increased at a rate of 3 °C/min up to 80 °C, followed by 5 °C/min up to 230 °C, and held for 6 min. The ionization voltage was 70 eV, and the mass range was m/z 40–350. A solvent delay was set at 6.0 min.

Volatile compounds were identified using the NIST 14 database. Peaks were quantified relative to the internal standard using the peak area of an extracted ion. For semi-quantification purposes, the relative peak area of each identified compound was measured and then compared with the relative peak area of the added internal standard [11,12]. Volatile compound analysis was performed in triplicate (*n* = 3). The authors of the paper are aware that this application did not conform to an accurate quantification; therefore, we did not compare the quantities to other compounds or other publications, but only within the same compound was a comparison allowed. For the interpretation of the data this presentation was sufficient and, above all, saved time and costs in method development.

### 2.5. Statistical Analysis

Statistical analysis was performed using SPSS 26 (SPSS Inc., Chicago, IL, USA). The data obtained were subjected to one-way analysis of variance, and the significance level was considered at *p* < 0.05. The physicochemical data were mainly processed using Origin (version 2018) (Origin Lab Inc., Hampton, MS, USA). The levels of variable importance for predictive components (VIP) for microorganisms and volatile compounds were calculated using SIMCA 14.1 software. The correlation heatmap is made by Heml1.0.3.7 software.

## 3. Results and Discussion

### 3.1. Dynamics of Physicochemical Properties during Fermentation

Table 2 shows the changes in physicochemical parameters, including ethanol, reducing sugar, total acids and pH, during fermentation with four different starters. The lowest content of reducing sugar was observed in wine made by F15 (0.63 g/L), followed by CECA-fermented liquor (0.7 g/L). On the second day of fermentation, CEC01 reduced the reducing sugar in must by 40.9%, and the initial fermentation rate was significantly faster than that of other starters, which was consistent with some reported results [13,14].

The total acid decreased from 6–7 g/L initially to 4.2–4.5 g/L at day 6, except for 796 (5.4 g/L), which was significantly higher than other samples. As for CEC01, a continuous decrease in total acid was observed until the end of the fermentation. This trend was different from the other starters, which was also reported previously [15,16]. It indicated that CEC01 plays an important role in reducing acid. The pH did not change obviously before and after fermentation, but CECA and CEC01 were observed to increase significantly on day 4 of fermentation.

### 3.2. Comparison of Microbial Diversity among Samples

HTS was used to identify the succession of the microbial community during the Cabernet Sauvignon fermentation. A total of 1,053,790 (average length = 354 bp) quality filtered sequencing reads corresponding to the ITS1F_ITS2R regions of fungal ITS rRNA genes were obtained. All sequences were clustered into 366 OTUs with 97% similarity (Supplementary Figure S1). These sequences were annotated into 299 fungal species. The alpha diversity indices, such as Chao, ACE, Shannon, Simpson and Good’s coverage, were used to determine microbial diversity (Supplementary Table S1). Good’s coverage of all samples reached 99%, indicating that the data coverage provided a satisfactory description of the microbial diversity.

**Figure 1:**
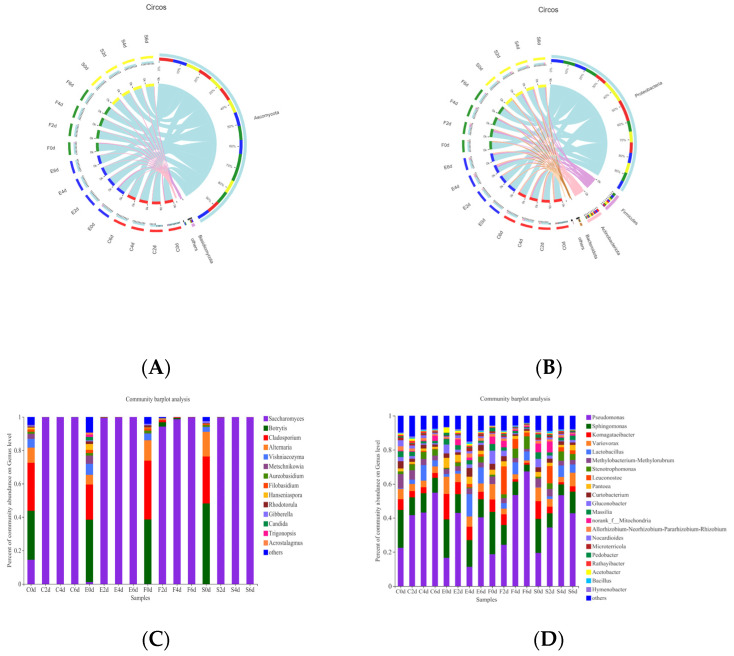
Microbiota composition. (**A**,**B**): The Circos graphs of fungi (**A**) and bacteria (**B**) at the phylum level in 20 samples. (**C**,**D**): Relative abundance of fungi (**C**) and bacteria **(D**) on the genus level.

**Figure 2:**
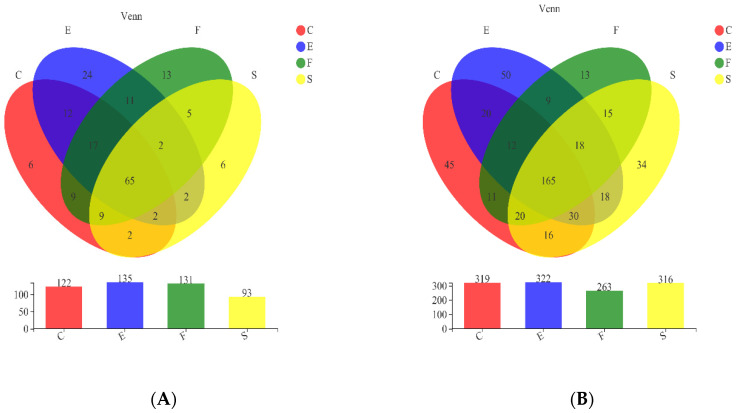
Venn diagram of (**A**) fungal and (**B**) bacterial genera among samples resulting from alcoholic fermentation. C: CECA; E: CEC01; F: F15; S: 796.

The dominant phylum across the entire eukaryotic population was *Ascomycota*, followed by *Basidiomycota* and other fungi. Moreover, the dynamics of microbial populations of all the samples at phylum level were very similar, but the relative abundances varied during the fermentation and among different starters (Figure 1A). The distribution of the fungal genus with relative abundance above 1% is presented in Figure 1C. Fourteen fungal genera were detected in sixteen samples. With respect to the overall proportion, *Saccharomyces*, *Botrytis*, *Cladosporium*, *Vishniacozyma* and *Alternaria* were the most prevalent fungal taxa throughout the fermentation. There were 135 fungal genera in fermentation by CEC01; among them, 24 genera were unique, which was higher than other starters, followed by F15 (Figure 2A). The lowest number of fungi was observed in the fermentation broth inoculated with 796. A total of 65 genera coexisted in the fermentation process of four strains. CECA and CEC01 shared the most genera in the must, with 12 genera.

*Botrytis* and *Cladosporium* were the predominant fungal genera in grape juice. They have also been found in grape skins and spontaneous fermentation [17,18]. After fermentation started, *S. cerevisiae* grew and rapidly dominated the community, while other species underwent drastic decreases in relative abundances during the fermentation. However, the relative abundance of Botrytis and Cladosporium in F2d was higher than that in other experimental groups, which was related to the slow start of fermentation by F15, and the inhibitory effect of lower alcohol concentration on microorganisms was weaker.

For bacteria diversity, all quality filtered sequences were clustered into 870 OTUs (97% similarity level) (Supplementary Figure S2), and 25 bacterial phyla were detected in alcoholic fermentation. The richness and diversity of the microbial communities in each sample were assessed by alpha diversity. The ACE and Chao diversity indices showed that the abundance of bacteria in the samples was remarkably higher than that of fungi (Supplementary Table S2). Concerning the prokaryotic communities, the dominant phyla were *Proteobacteria*, *Firmicutes*, *Actinobacteria* and *Bacteroidota* (Figure 1B). The members of underrepresented phyla were grouped together in the artificial group “Others”. A related study [19,20] indicated that the dominant phyla on the surface of wine grapes are *Proteobacteria*, *Actinobacteria* and *Firmicutes*. Most of the bacteria in the fermentation process of the sample may have come from the berry epidermis and vineyard environment [21]. As a reflection of the microbial community dynamics, the relative abundances of all prokaryotic communities varied in time and among different samples. *Firmicutes* (33.4%) was dominant in the fermentation process of F15; the dominant phylum in CECA was *Actinomycetes*. A total of 476 bacterial genera were detected in the 16 groups, of which 21 bacterial genera with relative abundances greater than 1% were selected for further analysis (Figure 1D). Similar to the results observed in fungi, CEC01 had the highest number of bacterial genera, with 322, followed by CECA with 319 genera (Figure 2B). Accordingly, the numbers of unique genera in fermentation by CEC01 and CECA were more than those of other strains, 50 and 45, respectively. The difference was that the lowest number of bacteria were observed in the fermentation broth added to F15. The number of bacteria in all samples were higher than that of fungi. A total of 165 genera of bacteria were found to be common in the fermentation process of the four starters.

*Pseudomonas*, *Sphingomonas*, *Variovorax*, *Lactobacillus*, and *Komagataeibacter* were the most prevalent bacteria in fermentation (Figure 1D). These were also reported to be the dominant genera of grape berries [22]. Overall, the relative abundance of *Pseudomonas* in all the samples at the end of fermentation was higher than that on day 0, while *Sphingomonas* showed the opposite succession rule. The abundance of *Pseudomonas* in E4d decreased to 11.2%, and then increased to 60.3% on day 6 of fermentation; the abundance of *Pseudomonas* in must with the 796 strain increased to 53.4% on day 4 and then decreased to 42.7% on day 6 of fermentation, but it was still higher than that of unfermented grape juice. *Sphingomonas* has also been detected in other grape mash and wine [23,24]. However, its effects on wine quality and sensory characteristics are still unknown. The relative abundances of *Gluconobacter* and *Komagataeibacter* in the middle and later stages of fermentation by the four strains were relatively low. This may be because the predominant commercial high-activity *Saccharomyces* certainly inhibited the growth of acid-producing bacteria in microflora [25,26,27]. The relative abundance of *Lactobacillus* increased obviously on day 4 for each fermented sample, and the highest abundance was found in E4d. This may be due to the release of nutrients such as vitamins and amino acids by the yeast, which promoted *Lactobacillus* growth during alcohol fermentation [28].

### 3.3. Change in Volatile Compounds during Fermentation

A total of 58 aroma compounds were identified in the samples, including 17 alcohols, 25 esters, 7 acids, 5 aldehydes and others. The odor descriptions of volatile compounds (Supplementary Table S3) referred to previous literature reports [2,29,30]. The esters represented the largest group in terms of the number in almost all wines, followed by alcohols and fatty acids. Most ester compounds, which could provide the fruity aroma characters desired in wine, are formed during fermentation, especially acetate and ethyl esters [31,32]. Acetate is formed with the higher alcohol production by the degradation of amino acids or sugars and acetyl coenzyme A [33]. In this experiment, 9 kinds of acetates were detected in the fermentation broth of different yeasts. Furthermore, 9 kinds of fatty acid ethyl esters were detected in this work. Fatty acid ethyl esters are produced by the reaction of ethanol with the fatty acid coenzyme A produced by the yeast fatty acid metabolism during alcohol fermentation [34]. It is also related to the content of fatty acids in the raw materials. The concentrations of Ethyl caprylate and Ethyl laurate initially increased and then decreased. This was consistent with the dynamics of octanoic acid and lauric acid during fermentation. Ethyl nonanoate could only be detected in the middle and late stages of fermentation inoculated with CEC01, which can give wine a strong grape flavor and rose aroma; nevertheless, the generated content was small and therefore not obvious. At the end of fermentation (Supplementary Table S3), Ethyl caprylate (banana, pear, flower), Ethyl caprate (pear, brandy), Ethyl caproate (apple, pineapple, banana), isoamyl acetate (banana) and Ethyl 9-decenoate (fruity) will bring pleasant floral and fruity aromas to the wine. This accords with previous reports [35].

Higher alcohols are produced by yeast-degrading amino acids through the Ehrlich pathway, and their contribution to wine aroma depends on the concentration [36]. In general, a pleasant aroma can be produced when its mass concentration is lower than 300 mg/L [37]. When the alcoholic fermentation finished, the total higher alcohol concentration of wine fermented by CECA was the highest, followed by that of F15. In particular, the content of phenethyl alcohol in C6d was significantly higher than the other samples in the same period, which may bring the aroma of roses and honey to the wine [38].

Volatile fatty acids in wine are mainly produced by yeast and lactic acid bacteria in the fatty acid metabolism, usually resulting in fruit, cheese, fat and sour tastes [39]. In this work, five fatty acids were detected in the wine samples after alcohol fermentation. Compared with the wine samples of the same period, the unique fatty acids in F6d were acetic acid and stearic acid, while trans-2-hexenoic acid was unique to S6d.

**Figure 3:**
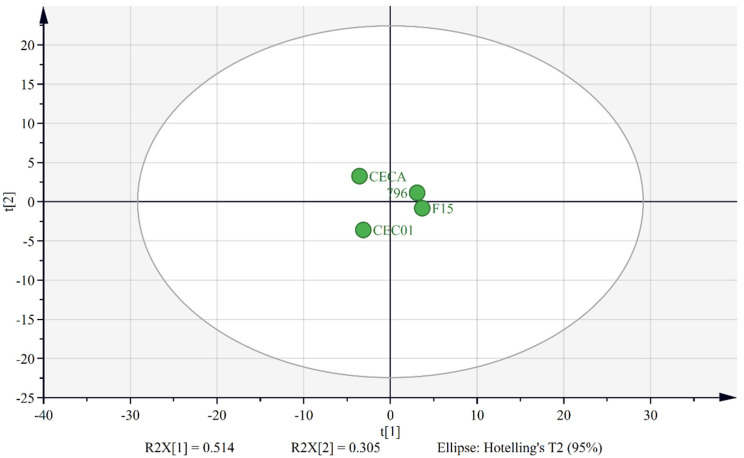
PCA score scatter plot of the volatile compounds in pineapple wines fermented by four yeast strains.

The similarity of the four wines was visualized by PCA (Figure 3), using the concentrations of the volatile compounds detected in the samples of the four starters on day 6 as analytical variables. The percentage of the cumulative contribution of the variance of the first two PCs was 81.9%. PC1 and PC2 represented 51.4 and 30.5% variabilities of the volatile compounds, respectively. The score scatter plot shows that the four samples were situated in four quadrants. The wine fermented by the yeasts 796 and F15 formed a single cluster, suggesting that these two yeasts produced wine with a similar aroma profile. The wine samples of CECA and CEC01 were distributed in the second and third quadrant, respectively, and these two samples were relatively far away from each other in the plot, indicating that a difference in volatile aroma between them existed.

### 3.4. Correlation Analysis between Microbiota and Volatile Compounds

The varieties of the microbial consortium in the fermentation can lead to different characteristics of wine products. As the microbial metabolisms, the volatile aroma components are closely linked to the metabolic activities of some microorganisms. Thus, Spearman’s correlation coefficients between the detected aroma components and core microorganisms were calculated to preliminarily indicate the causal relationships between them. The core microbial taxa with VIP > 1 (the top 50 microorganisms as variables) were selected from the fermentation stages of each starter by constructing the PLS-DA model.

**Figure 4:**
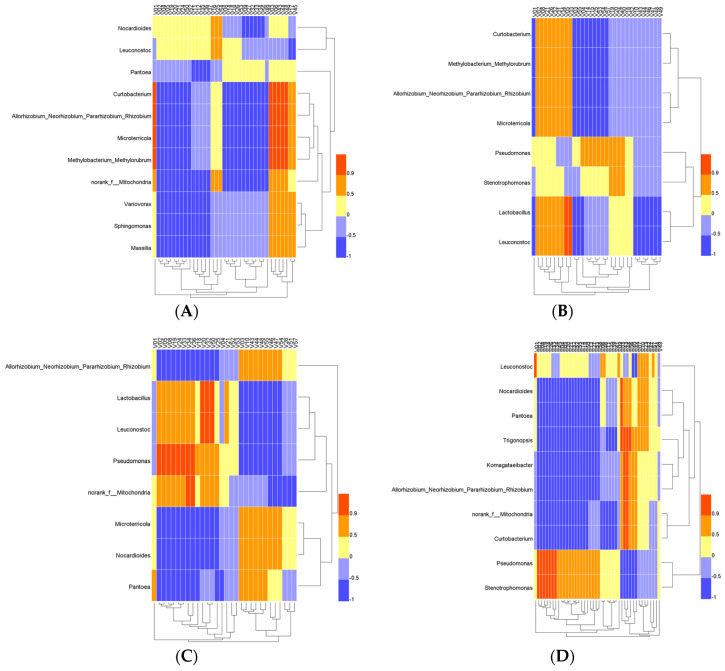
Heatmap of core microbiota and volatile compounds in alcoholic fermentation stages by different commercial starters. V01–V58: volatile compounds. Spearman’s correlation coefficients (|ρ| > 0.7) and *p* values are identified. (**A**) 796; (**B**) CEC01; (**C**) CECA; (**D**) F15.

The correlation between microbes and metabolites is shown in Figure 4. A total of 11, 8, 8, 10 microbial genera were identified as core microorganisms in the fermentation of 796, CEC01, CECA, F15 starter, respectively. Figure 4A shows that four microbial genera (*Curtobacterium, Allorhizobium_Neorhizobium_Pararhizobium_Rhizobium, Microterricola, Methylobacterium_Methylorubrum*) in the 796 fermentative process strongly positively correlated with the following volatile compounds: 1-Hexanol (V01), 3-Hexen-1-ol (V03), Terpinen-4-ol (V06), Hexanal (V43), trans-2-Hexenal (V44), and 3-Octanone (V48). *Leuconostoc* correlated positively with the following substances: Lauryl alcohol (V16), Benzyl acetate (V25), and Hexanoicacid (V54). *Variovorax*, *Sphingomonas* and *Massilia* correlated positively with the following substances: 3-Hexen-1-ol (V03), Terpinen-4-ol (V06), Hexanal (V43), trans-2-Hexenal (V44), 3-Octanone (V48), cis-6-Nonen-1-ol (V17), and Benzaldehyde (V45).

*Leuconostoc* and *Lactobacillus* in the fermentation of CEC01 (Figure 4B) were strongly positively correlated with the following compounds: Phenylethyl alcohol (V05) and Phenethyl acetate (V33), and also positively correlated with the following substances: Isopentanol (V02), Isobutanol (V08), Ethyl laurate (V21), Isoamyl acetate (V26), Ethyl-9 decanoate (V32), Ethyl nonanoate (V31), Ethyl butyrate (V34). *Pseudomonas* correlated positively with the following substances: Benzyl alcohol (V04), 1-Octanol (V14), 1-Decanol (V15), Benzyl acetate (V25), Decanoic acid (V53), Hexanoic acid (V54), Lauric acid (V57), Ethyl caprylate (V19), Ethyl caprate (V20), Ethyl caproate (V24), and Styrene (V50).

*Leuconostoc* and *Lactobacillus* in the fermentation of CECA (Figure 4C) were strongly positively correlated with the following compounds: Ethyl caprate (V20), Ethyl-9-decenoate (V32), and Styrene (V50), and also positively correlated with the following substances: Isopentanol (V02), Phenylethyl alcohol (V05), Isobutanol (V08), Ethyl caprylate (V19), Ethyl caproate (V24), Phenethyl acetate (V33), Ethyl butyrate (V34), Isopentyl hexanoate (V36), and Ethyl laurate (V21). *Pseudomonas* correlated strongly positively with the following substances: Isopentanol (V02), Phenylethyl alcohol (V05), Isobutanol (V08), Ethyl caprylate (V19), Ethyl caproate (V24), Phenethyl acetate (V33), Ethyl butyrate (V34), and Isopentyl hexanoate (V36), and also positively correlated with the following substances: Hexyl acetate (V18), Ethyl caprate (V20), Ethyl-9-decenoate (V32), Styrene (V50), and Benzyl acetate (V25). *Norank_f__Mitochondria* correlated strongly positively with the following substances: Ethyl butyrate (V34) and Isopentyl hexanoate (V36).

There was a strong positive correlation between *Leuconostoc* and 1-hexanol in the fermentation of F15 (Figure 4D). *Pseudomonas* and *Stenotrophomonas* both had a strong positive correlation with the following substances: Isopentanol (V02), Isobutanol (V08), Ethyl caprylate (V19), Ethyl caproate (V24), Isoamyl acetate (V26), Phenethyl acetate (V33), and Ethyl butyrate (V34).

*Lactobacillus* and *Leuconostoc* in wines degrade both citric acid and malic acid; therefore, they significantly affect the taste of wine and increase some aromas with a flavor complexity [40], indicating that the potential to produce aromatic substances by glycosidase activity [41] was a common core microorganism in the fermentation of 796, CEC01, CECA and F15. *Lactobacillus* only appeared in the core microflora of CEC01 and CECA. According to the correlation data, *Lactobacillus* and *Leuconostoc* promoted the production of phenylethyl alcohol and phenylethyl acetate in the fermentation process of CEC01, which may bring a rose fragrance to the final wine [42]. *Pseudomonas* is one of the most common bacteria in soil, which can also be separated from wine cork. It is related to the pyrazine compounds with a low sensory threshold, which may have a negative impact on wine flavor [43]. Pyrazine compounds were not detected in any samples in this experiment. *Pseudomonas* was a common core microorganism in the fermentation of CEC01, CECA and F15. There was a strong positive correlation between *Pseudomonas* and phenethyl acetate in the wine samples of CECA and F15. *Sphingomonas* was a unique core microorganism in the fermentation of 796. *Sphingomonas* can degrade carbon sources at an incubation temperature of 31 °C [44]. Earlier reports determined that *Sphingomonas* positively correlated with fermentation rate [45], and *Sphingomonas* can degrade aromatic compounds, which may lead to decreased methyl caprate and 4-ethylphenol [46]. In 796 fermented samples, this microorganism may have been related to the formation of such volatile substances as 3-Hexen-1-ol, Hexanal and 3-Octanone.

In summary, the core microorganisms of different starters were different in the process of alcohol fermentation. Some microbes, such as certain bacteria, may have come from the vineyard environment. Although *Saccharomyces cerevisiae* played a dominant role in the fermentation process, there were still some strains that may antagonize or cooperate with environmental microorganisms to inhibit or promote the synthesis of some aroma components [47]. The compositions of the core fermentation microorganisms of CECA and CEC01 were similar. The compositions of the core fermentation microorganisms of F15 and 796 were similar. This agreed with the PCA result of volatile aroma.

## 4. Conclusions

Different starters affected the aroma and microbial succession of Cabernet Sauvignon wine. With the advance of the fermentation process, the microbial abundance of different starter samples was different, showing an overall downward trend. Saccharomyces cerevisiae significantly inhibited the growth of other microorganisms. Though the bacterial community composition of each sample was similar, the relative abundance was different. *Pseudomonas*, *Sphingomonas*, *Variovorax*, *Lactobacillus*, and *Komagataeibacter* were the most prevalent bacteria in fermentation. For aroma component, there was no significant difference in the composition of aroma species produced by different starters, but there were significant differences in the correlation between the microorganisms and volatile aroma of wine made by different starters, and the contribution of the same core microorganism to aroma in different samples was also different. This indicated that the type of starter will have an effect on the flavor quality of wine, and different starters have different adaptabilities to wines of specific varieties in specific producing areas. The results provide theoretical support for shaping the characteristics and flavor typicality of wine-producing areas. With regard to volatiles, the results of this present study should be verified in further investigations, with a view to accurate quantification.

## Figures and Tables

**Table 1:** Description of the acronyms used for the samples of this study.

Fermentation Time	CECA	CEC01	F15	796
Day 0	C0d	E0d	F0d	S0d
Day 2	C2d	E2d	F2d	S2d
Day 4	C4d	E4d	F4d	S4d
Day 6	C6d	E6d	F6d	S6d

**Table 2:** General physicochemical properties of the wines fermented by four yeast strains. Values shown represent averages of triplicate samples (data are mean ± SD). Values with different superscript roman letters in the same row are significantly different according to the Duncan test (*p* < 0.05).

Starters	Fermentation Days	Reducing Sugar (g/L)	Ethanol (% vol)	pH	Total Acid (g/L)
CECA	Day 0	221.33 ± 0.93 ^a^	0	3.71 ± 0.01 ^a^	6.23 ± 0.06 ^a^
	Day 2	177.23 ± 2.24 ^b^	2.01 ± 0.04 ^b^	3.80 ± 0.01 ^a^	4.00 ± 0.01 ^b^
	Day 4	44.00 ± 0.56 ^c^	10.47 ± 0.02 ^a^	3.89 ± 0.006 ^a^	4.13 ± 0.06 ^b^
	Day 6	0.70 ± 0.20 ^d^	12.37 ± 0.04 ^a^	3.82 ± 0.006 ^a^	4.20 ± 0.02 ^b^
CEC01	Day 0	224.77 ± 0.06 ^a^	0	3.67 ± 0.006 ^a^	6.57 ± 0.06 ^a^
	Day 2	132.73 ± 1.16 ^b^	5.21 ± 0.03 ^b^	3.72 ± 0.006 ^a^	4.63 ± 0.06 ^b^
	Day 4	27.50 ± 0.44 ^c^	11.74 ± 0.04 ^a^	3.79 ± 0.006 ^a^	4.50 ± 0.01 ^b^
	Day 6	1.00 ± 0.26 ^d^	12.67 ± 0.01 ^a^	3.75 ± 0.006 ^a^	4.30 ± 0.01 ^b^
F15	Day 0	225.40 ± 0.20 ^a^	0	3.66 ± 0.01 ^c^	6.56 ± 0.06 ^a^
	Day 2	212.50 ± 1.36 ^b^	0.036 ± 0.02 ^d^	3.68 ± 0.1 ^b^	3.60 ± 0.02 ^d^
	Day 4	64.63 ± 1.36 ^c^	9.17 ± 0.01 ^c^	3.68 ± 0.006 ^b^	4.56 ± 0.06 ^b^
	Day 6	0.63 ± 0.12 ^d^	12.66 ± 0.02 ^b^	3.72 ± 0.002 ^a^	4.53 ± 0.06 ^b^
796	Day 0	222.00 ± 0.89 ^a^	0	3.69 ± 0.06 ^b^	6.93 ± 0.06 ^a^
	Day 2	170.43 ± 2.00 ^b^	2.19 ± 0.09 ^d^	3.72 ± 0.01 ^a^	4.53 ± 0.06 ^d^
	Day 4	51.03 ± 0.93 ^c^	9.64 ± 0.01 ^c^	3.73 ± 0.06 ^a^	5.57 ± 0.06 ^c^
	Day 6	1.17 ± 0.06 ^d^	12.41 ± 0.02 ^b^	3.74 ± 0.06 ^a^	5.73 ± 0.06 ^b^

## Data Availability

All data stemming from the present research are enclosed in the tables or as Supplementary Materials. Any additional data will be made accessible from the corresponding authors upon reasonable request.

## Supplementary Materials

The following supporting information can be downloaded at: https://www.mdpi.com/article/10.3390/foods11121725/s1, Figure S1: Dilution curve of Shannon index of fungi on OUT level; Figure S2: Dilution curve of Shannon index of bacteria on OUT level; Table S1: Alpha diversity indices of Fungus; Table S2: Alpha diversity indices of bacteria; Table S3: Semi-quantitative concentration of the volatile compounds (ratio among peak area of volatile compounds and internal standards) produced from the alcohol fermentation by different starters.
