# Supplementary material for: Effect of Commercial Yeast Starter Cultures on Cabernet Sauvignon Wine Aroma Compounds and Microbiota
Source: Foods. 2022 Jun 13;11(12):1725. doi: 10.3390/foods11121725 (PMC9222704; doi:10.3390/foods11121725)
Supplement: Supplementary file 1 [file foods-11-01725-s001.zip › Supplementary Figures.pdf]

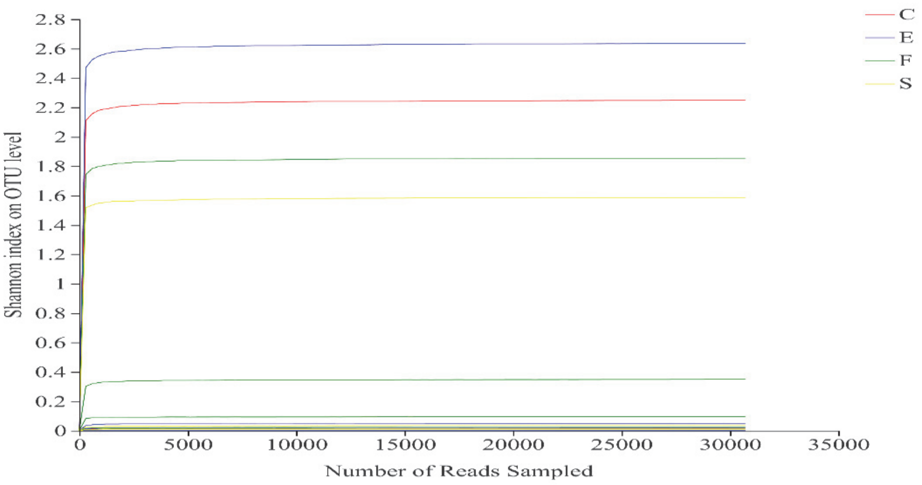

Supplementary Figure S1. Dilution curve of Shannon index of fungi on OUT level. C: CECA; E: CEC01; F: F15; S: 796.

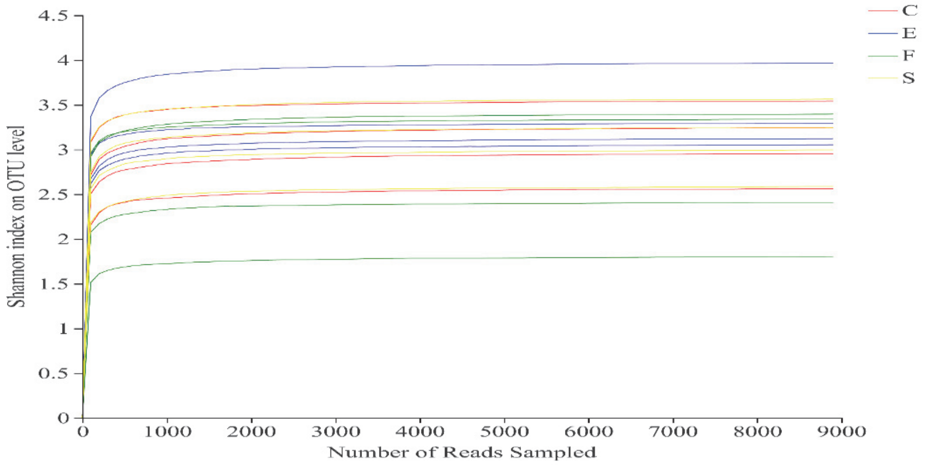

Supplementary Figure S2. Dilution curve of Shannon index of bacteria on OUT level. C: CECA; E: CEC01; F: F15; S: 796.
