# Supplementary material for: Effect of Commercial Yeast Starter Cultures on Cabernet Sauvignon Wine Aroma Compounds and Microbiota
Source: Foods. 2022 Jun 13;11(12):1725. doi: 10.3390/foods11121725 (PMC9222704; doi:10.3390/foods11121725)
Supplement: Supplementary file 1 [file foods-11-01725-s001.zip › Supplementary Table.pdf]

Supplementary Table S1

Alpha diversity indices of Fungus.

Sample	Alpha diversity indices				
	Chao	ACE	Shannon	Simpson	Coverage
C0d	137.25	135.64	2.036	0.202	0.999
C2d	22.50	40.00	0.014	0.997	0.999
C4d	24.20	28.45	0.014	0.997	0.999
C6d	8.00	10.08	0.004	0.999	0.999
E0d	147.61	145.19	2.334	0.198	0.999
E2d	41.60	62.60	0.047	0.989	0.999
E4d	21.20	23.31	0.017	0.996	0.999
E6d	11.00	0	0.001	0.999	0.999
F0d	132.37	135.79	1.657	0.290	0.999
F2d	106.80	130.78	0.337	0.889	0.999
F4d	43.60	70.11	0.094	0.973	0.999
F6d	22.25	25.88	0.022	0.995	0.999
S0d	121.36	118.08	1.436	0.334	0.999
S2d	44.50	67.98	0.033	0.992	0.999
S4d	20.00	43.69	0.011	0.998	0.999
S6d	30.00	40.72	0.005	0.999	0.999

Supplementary Table S2

Alpha diversity indices of bacteria.

Sample	Alpha diversity indices				
	Chao	ACE	Shannon	Simpson	Coverage
C0d	244.12	301.10	2.80	0.121	0.993
C2d	293.13	363.26	2.73	0.195	0.992
C4d	248.12	300.84	2.47	0.215	0.993
C6d	251.55	325.96	2.16	0.316	0.992
E0d	183.33	281.89	2.59	0.122	0.994
E2d	265.04	334.21	2.50	0.210	0.992
E4d	323.89	391.44	3.29	0.074	0.991
E6d	219.75	244.85	2.56	0.192	0.994
F0d	230.00	286.71	2.62	0.125	0.994
F2d	270.60	368.32	2.88	0.112	0.992
F4d	228.00	335.40	2.07	0.306	0.993
F6d	201.56	249.90	1.58	0.463	0.994
S0d	280.90	357.83	2.82	0.107	0.992
S2d	265.36	317.75	2.78	0.148	0.992
S4d	263.66	332.29	2.24	0.300	0.992
S6d	278.26	367.62	2.42	0.213	0.992

Supplementary Table S3

Semi-quantitative concentration of the volatile compounds (ratio among peak area of volatile compounds and internal standards) produced from the alcohol fermentation by different starters. Data are given as means ± SD (n = 3). Values with different letters in the same row are significantly different ($P < 0.05$) from each other. ND: not detected.

	Compounds (10^{-5})	Odor description [2,29,30]	Numbered	Starters			
				S6d	F6d	C6d	E6d
Alcohol (10)	Hexyl alcohol	Herbaceous, grass	V01	41.51±1.14[a]	39.46±1.62[a]	24.24±2.01[b]	25.77±0.92[b]
	isoamyl alcohol	Alcohol, harsh, bitter, banana	V02	501.92±2.26[c]	586.90±1.82[b]	951.86±24.03[a]	377.81±10.23[d]
	Benzyl alcohol	Almond, flowery	V04	2.76±0.12[c]	1.38±0.30[d]	6.47±0.49[a]	ND
	Phenethyl alcohol	Flowery-rose, bitter, fruity-peach	V05	210.04±0.10[d]	164.13±1.95[e]	719.93±18.68[a]	330.19±33.01[b]
	2-Methyl-1-propanol	Spirituous, fuel	V08	15.20±0.33[c]	30.12±0.28[b]	55.5±2.87[a]	14.26±0.69[c]
	n-heptanol	Chemical, green	V09	2.72±0.27[a]	1.51±0.17[b]	ND	ND
	3-Methyl-1-pentanol	Brandy, dirt, green fruit	V12	ND	0.95±0.24	ND	ND
	1-Octanol	Chemical, metal, burnt	V14	3.57±0.21[b]	3.98±0.11[a]	ND	ND

	**Total amount**			777.72±3.23[cd]	828.46±5.14[c]	1758.00±43.01[a]	748.04±42.74[d]
Ester (14)	Hexyl acetate	Apple, cherry, pear, fmoral	V18	36.15±4.53[b]	30.73±1.39[b]	21.86±1.58[bc]	10.23±2.56[c]
	Ethyl caprylate	Fruity, pineapple, pear	V19	640.87±4.63[b]	422.00±22.21[d]	586.00±19.73[c]	265.33±5.28[e]
	Ethyl caprate	Grape, floral	V20	226.58±5.46[b]	154.59±3.27[c]	235.86±17.63[b]	110.19±0.5133[d]
	Ethyl laurate	Leaf	V21	31.93±2.29[c]	61.32±4.62[b]	21.67±1.95[d]	20.25±3.36[d]
	Ethyl caproate	Apple peel, fruit	V24	238.93±21.98[a]	151.37±1.51 [c]	143.77±10.08[c]	70.11±3.45[d]
	Benzyl acetate	Flowery-jasmine	V25	ND	ND	0.19±0.14	ND
	Isoamyl acetate	Fruity, sweet, banana	V26	160.91±1.84[b]	167.13±4.72[b]	ND	65.48±0.78[c]
	Ethyl acetate	Pineapple, fruity	V27	76.17±9.43[a]	74.77±2.28[a]	ND	ND
	Ethyl nonanoate	Floral, fruity	V31	ND	ND	ND	0.98±0.17
	Ethyl 9-decenoate	Rose, Green, fruity, fatty	V32	39.19±1.49[a]	22.42±2.81[a]	4.38±0.31[a]	6.76±0.76[a]
	Phenethyl acetate	Apple, cherry, pear, fmoral1	V33	25.27±3.74[c]	27.07±3.05[c]	73.41±6.53[a]	15.83±0.62[d]

	Ethyl butyrate	Pineapple, yellow passion fruit	V34	16.16±1.11[a]	7.05±0.7[b]	4.42±1.46[c]	2.11±0.63[d]
	Isopentyl hexanoate	Apple, pineapple	V36	ND	ND	233.84±9.31a	ND
	**Total amount**			1492.53±42.00[b]	1118.46±36.75[d]	1325.40±68.71[c]	567.27±16.54[e]
Acid (5)	Octanoic acid	Sweat, cheese	V52	28.24±2.39[ab]	31.24±2.12[a]	22.17±2.99[c]	8.13±0.80[d]
	Decanoic acid	Rancid, fat	V53	28.39±0.98[a]	15.79±1.36[b]	6.41±2.45[c]	ND
	Acetic acid	Sour	V55	ND	1.43±0.45	ND	ND
	trans-2-Hexenoic acid	Pineapple, hawthorn, strawberry, cheese	V54	1.47±0.39	ND	ND	ND
	Stearic acid	Fat	V58	ND	4.02±0.28	ND	ND
	**Total amount**			58.11±1.07[a]	52.49±4.06[a]	28.59±5.44[b]	8.13±0.80[c]
others(4)	Benzaldehyde	Almond, burnt sugar	V45	ND	1.86±0.69	ND	ND
	2,4-Di-tert-butylphenol		V49	5.12±1.43[a]	6.69±1.03[a]	ND	ND
	Styrene	Balsamic, gasoline	V50	6.33±1.15[a]	7.05±0.96[a]	4.87±0.17[b]	4.80±0.24[b]
	**Total amount**			11.45±0.29[b]	15.61±2.66[a]	4.87±0.17[c]	4.80±0.24[c]
